# Structural alteration of a BYDV-like translation element (BTE) that attenuates p35 expression in three mild *Tobacco bushy top virus isolates*

**DOI:** 10.1038/s41598-017-04598-5

**Published:** 2017-06-23

**Authors:** Deya Wang, Chengming Yu, Shanshan Liu, Guolu Wang, Kerong Shi, Xiangdong Li, Xuefeng Yuan

**Affiliations:** 1 Department of Plant Pathology, College of Plant Protection, Shandong Agricultural University, Tai’an, 271018 P. R. China; 2 College of Animal Science and Veterinary Medicine, Shandong Agricultural University, Tai’an, 271018 P. R. China

## Abstract

To identify the molecular effects of *Tobacco bushy top virus* (TBTV) evolution on the degeneration of tobacco bushy top disease, three TBTV isolates with mild virulence were compared with wild-type TBTV to assess the translation of p35, which relies on a BYDV-like translation element (BTE) in a cap-independent manner. The *in vitro* expression of p35 in the mild isolates was only 20% to 40% of the expression observed in wt TBTV. Based on translation data from chimeric TBTV RNA, low-level p35 expression in the three mild isolates was associated with two regions: the 5′ terminal 500 nt region (RI) and the 3′ internal region (RV), which included the BTE. For the RV region, low level p35 expression was mainly associated with structural alterations of the BTE instead of specific sequence mutations within the BTE based on SHAPE structural probing and mutation analysis. Additionally, structural alteration of the TBTV BTE resulted from mutations outside of the BTE, implying structural complexity of the local region surrounding the BTE. This study is the first report on the structural alteration of the 3′ cap-independent translation element among different isolates of a given RNA virus, which is associated with variations in viral virulence.

## Introduction

The rapid life cycles of viruses require effective expression of viral proteins using the host translation machinery. The 5′ m7GpppN cap of most eukaryotic mRNAs recruits the ribosome by binding to numerous eukaryotic initiation factors (eIFs), such as eIF4E and eIF4G^1–3^. eIF4G also interacts with the 3′ poly(A)-binding protein (PABP) to circularize the mRNA, which may facilitate reinitiation using post-termination ribosomes^2, 4, 5^. The effective initiation and cyclic utilization of ribosomes ensures a high translation efficiency.

Many RNA viruses lacking a 5′ cap and/or 3′ poly(A) tail have evolved non-canonical mechanisms to recruit ribosome for the translation of viral proteins^6^. These non-canonical translation mechanisms mainly involve two types of cis-elements: 5′ internal ribosome entry sites(IRESs) and 3′ cap-independent translation enhancers(CITEs), which both possess diverse secondary structures to recruit ribosomes directly or via the assistance of a subset of eIFs^7, 8^. The 3′ CITEs have been predominantly characterized in plant viruses, including members of the family *Tombusviridae* and the genus *Luteovirus* of the family *Luteoviridae* ^6, 8, 9^. Furthermore, similar translational enhancers have also been identified in human transcripts^10^. The 3′ CITEs in plant RNA viruses have been classified into several typical types based on their origins, secondary structures and ability to bind to eIFs or ribosomes as follows: the translation enhancer domain (TED) derived from *satellite tobacco necrosis virus* (sTNV)^11^; the BYDV-like translation element (BTE) derived from *Barley yellow dwarf virus* (BYDV)^12^; the PMV-like translation element (PTE) derived from *Panicum mosaic virus* (PMV)^13^; the tRNA-like T-shaped structure (TSS) derived from *Turnip crinkle virus* (TCV)^14^; the large Y-shaped structure derived from *Tomato bushy stunt virus* (TBSV)^15^; the I-shaped structure derived from *Melon necrotic spot virus* (MNSV)^16^; and the kissing-loop T-shaped structure (kl-TSS) derived from *Pea enation mosaic virus* 2 (PEMV-2)^17^. To the best of our knowledge, RNA viruses undergo rapid evolution under natural selection with a higher mutation rate (10^−3^–10^−5^) due to error-prone replication in infected cells and organisms^18, 19^. Although different 3′ CITEs have been identified from various RNA viruses, few studies have focused on the structural variations of these 3′ CITEs among different isolates of given RNA virus.

*Tobacco bushy top virus* (TBTV), which is a member of the genus *Umbravirus* in the family *Tombusviridae*, and *Tobacco vein distorting virus* (TVDV), which is a member of the genus *Polerovirus* in the family *Luteoviridae*, were causal agents of tobacco bushy top disease in sub-Saharan Africa in the 1960s^20^ and in Asia, including China, in the 1990s^21^. Although aphid transmission of TBTV requires the presence of TVDV and its coat protein (CP), TBTV is independently pathogenic^21^. TBTV has a positive single-strand RNA genome of 4152 nt lacking a 5′ cap structure and a 3′ poly (A) tail^22^. TBTV encodes four ORFs that encode the replicase components including p35 and its −1 frameshift product p98 (the RdRp)^23, 24^ and the movement proteins p26 and p27^25, 26^. TBTV contains a functional BTE in its 3′ UTR that can regulate the cap-independent translation of a reporter gene^27^. Tobacco bushy top disease has suddenly appeared at 1990s in Yunnan province of China, and during the past decade the previously severe symptoms are currently more attenuated in the field^24^. During TBTV infection, the symptom could be associated with translation of the viral proteins, replication of the viral genome, virus movement and so on. In this study, the emphasis was focused on the relationship and mechanism between the differential expression of p35 and TBTV virulence in different isolates. cDNA clones of three TBTV isolates showing mild virulence in the field were constructed, and the cap-independent translation of p35 was analyzed *in vitro*. The p35 expression level of these mild isolates was only 20% to 40% of wild-type TBTV-Ch (TBTV China isolate in this study). This low level expression was partially associated with structural alterations of BTE instead of specific mutations within BTE.

## Results

### BTE plays an essential role in the cap-independent translation of p35 and *in vivo* accumulation of TBTV

**Figure 1:**
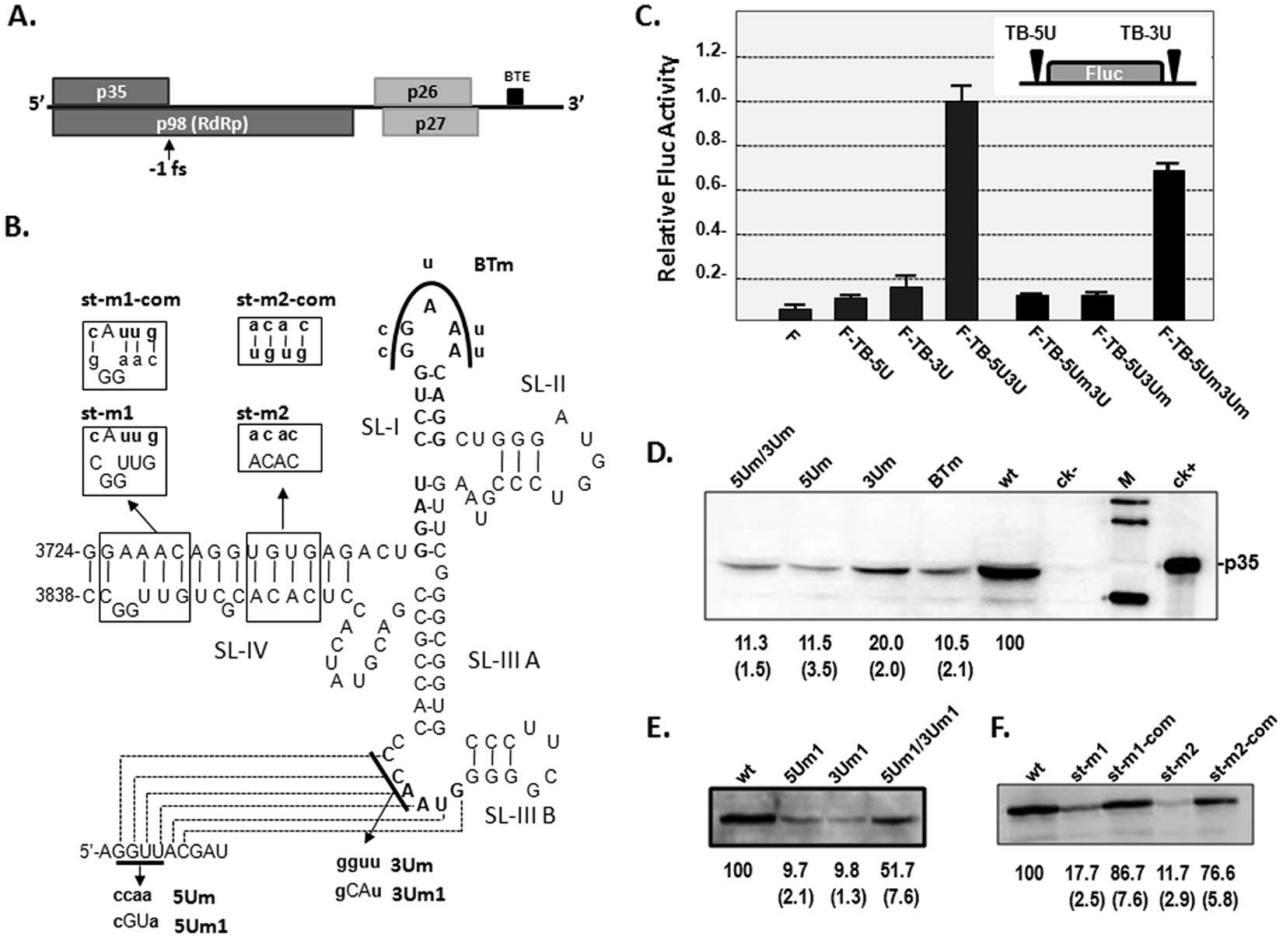
The effect of the BTE on translation of the fire luciferase reporter gene or p35 in TBTV. (**A**) Genome organization of the TBTV. RdRp: RNA-dependent RNA polymerase, BTE: Barley yellow dwarf virus (BYDV)-like translation elements, fs: frameshift. (**B**) Secondary structure of the BTE and putative RNA-RNA interactions with the 5′ UTR in TBTV. Bold nucleotides indicating the 17 nt consensus sequences of the BTE and the loop sequences that base-pair with the 5′ UTR; the broken polyline indicates potential base pairing, SL: stem-loop. (**C**) The effect of the BTE on Fluc translation. WGE: wheat germ extract. (**D**), (**E**) and (**F**) The effect of the BTE on p35 translation in full-length TBTV. wt: wild type TBTV (TBTV-ch),ck−: mock, ck+: purified p35, M: western blotting marker. The numbers under the western blots indicate the relative p35 level, and the numbers in bracket indicate the STEV.

The BTE of TBTV was reported to enhance the translation of reporter genes^27^. In this study, we firstly confirmed the translational enhancement properties of the TBTV BTE using a Fluc reporter construct (Fig. 1A,B,C). A Fluc construct containing the TBTV 5′ and 3′ UTRs (F-TB-5U3U) increased Fluc translation by ~20-fold, whereas Fluc constructs containing only the 5′ UTR or 3′ UTR of TBTV (F-TB-5U or F-TB-3U, respectively) had low level Fluc expression (Fig. 1C). Analogous to most other 3′ CITEs, the 5′ and 3′ UTRs (including the BTE) may enhance translation synergistically through a long-distance RNA-RNA interaction between the 5′ UTR and the BTE (Fig. 1B). Mutations (F-TB-5Um3U or F-TB-5U3Um) that disrupted this potential RNA-RNA interaction decreased translation of Fluc to 10% of F-TB-5U3U, whereas a compensatory mutation (F-TB-5Um3Um) that reformed this potential RNA-RNA interaction restored the translation of Fluc to ~70% of F-TB-5U3U (Fig. 1C).Thus, the RNA-RNA interaction between the 5′ UTR and the SL-IIIA loop within the BTE mediated the synergistic enhancement of Fluc synthesis.

To evaluate the function of the BTE in the full-length TBTV genome, corresponding mutations were constructed in a full-length cDNA clone of TBTV-Ch (wild-type TBTV) and tested for p35 expression. Mutations in the SL-I loop (BTm) or RNA-RNA interactions between the 5′ UTR and the BTE (5Um or 3Um) produced ~10% to 20% of the wt p35 expression level (Fig. 1B,D), indicating the importance of the interacting sequences and 17 nt conserved sequences in SL-I. However, the 5Um/3Um mutants containing compensatory mutations failed to restore p35 expression(Fig. 1D), possibly indicating the complexity of this long-distance RNA-RNA interaction between the 5′UTR and the BTE in the TBTV genome. A second set of compensatory mutations (5 Um1, 3 Um1 and 5 Um1/3 Um1) partially restored p35 translation, revealing the essential role of the RNA-RNA interaction between the 5′UTR and the BTE in p35 translation (Fig. 1B and E). Mutations in SL-IV in the BTE were also constructed to evaluate the importance of BTE structural stability for p35 translation. Mutations disrupting the SL-IV stem (st-m1 or st-m2) decreased p35 translation to 18% or 12% of the wt level, whereas compensatory mutations that reformed the stem (st-m1-com or st-m2-com) restored p35 expression to 87% or 77% of the wt level, respectively (Fig. 1B and F).This finding suggests that the SL-IV stem within the BTE is also important for p35 expression through stabilizing the BTE structure. Therefore, BTE stability plays an essential role in the cap-independent expression of p35 in the TBTV with synergistic regulation of the 5′UTR through a long-distance RNA-RNA interaction.

**Figure 2:**
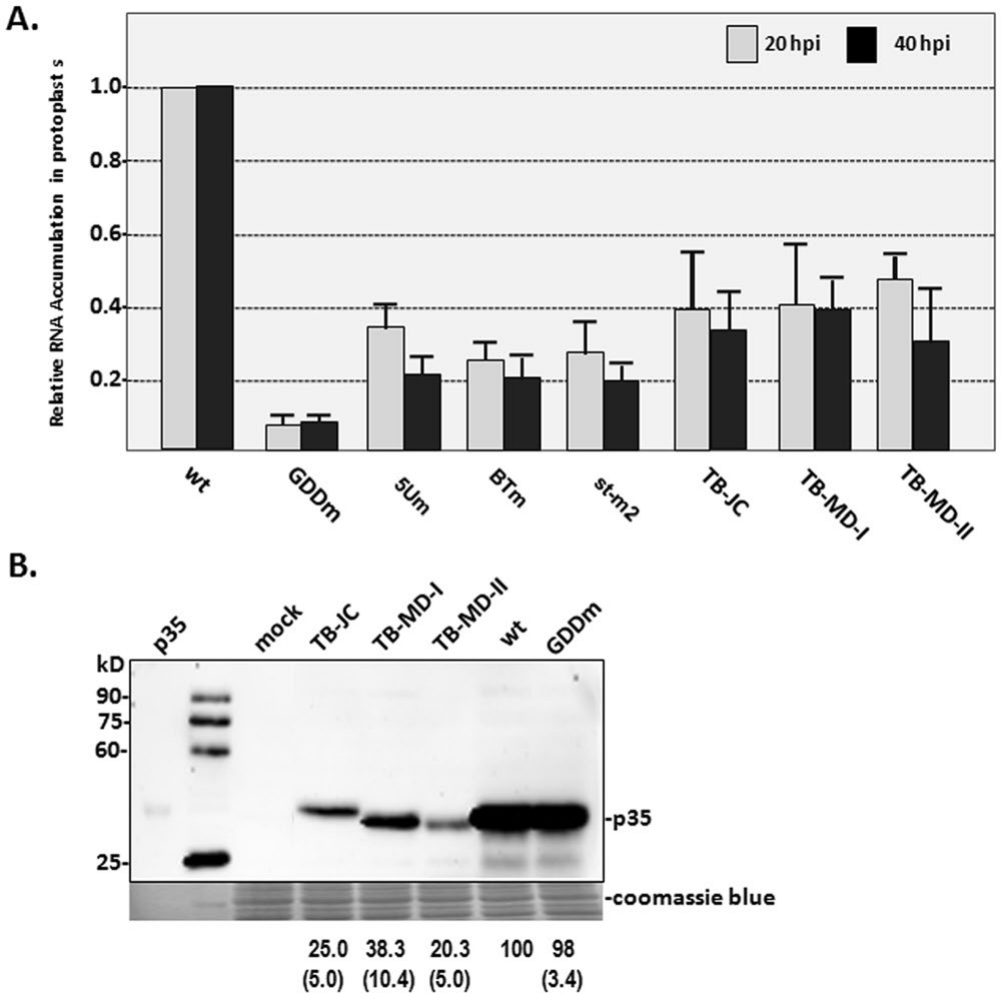
The relative RNA accumulation and p35 expression levels in different TBTV isolates and derivatives. (**A**) RNA accumulation of different TBTV isolates in protoplasts from BY-2. (**B**) p35expression in different TBTV isolates in WGE. Note: The antibody used in western blotting is p35 antiserum. wt: TBTV-Ch in this study; GDDm: GDD mutant of TBTV-Ch; TB-JC: TBTV Jiangchuan isolate; TB-MD-I: TBTV Midu-I isolate; TB-MD-II: TBTV Midu-II isolate. The numbers under the western blots indicate the relative p35 level, and the numbers in bracket indicate the STEV.

The role of BTE on *in vivo* accumulation of TBTV was also identified by analyzing the relative RNA accumulation of mutants associated with BTE region in protoplasts. Mutation (5 Um) disrupting the long-distance RNA-RNA interaction between BTE and 5′UTR decreased RNA accumulation to 35% at 20 hpi and 21% at 40 hpi of wt (Fig. 2A). Mutation on 17 nt conserved sequences in the SL-I loop (BTm) decreased RNA accumulation to 25% at 20 hpi and 20% at 40 hpi of wt (Fig. 2A). Mutation disrupting the stability of BTE (st-m2) decreased RNA accumulation to 27% at 20 hpi and 19% at 40 hpi of wt (Fig. 2A). These data suggests that the stable BTE and its associated long-distance RNA-RNA interaction is essential for *in vivo* accumulation of TBTV through regulating the cap-independent translation of p35.

### Low-level p35 expression in isolates exhibiting mild virulence is mainly associated with the 5′ terminal 500 nt region (RI) and 3′ internal region containing the BTE (RV)

Recently, tobacco bushy top disease has evolved based on the symptom differences in diseased plants in the field. Three mild isolates of TBTV (the JC, MD-I and MD-II isolates) were cloned from samples exhibiting mild virulence^24^. Their RNA accumulation was compared with TBTV-Ch (wt) in protoplasts from BY-2. The relative RNA accumulation of these mild isolates was only 40 to 47% of the wt at 20 hpi and 30 to 40% of the wt at 40 hpi (Fig. 2A), and the *in vitro* synthesis of p35 was 25%, 38% and 20% of the wt level for TB-JC, TB-MD-I and TB-MD-II, respectively (Fig. 2B). These findings suggest that the mild virulence of these new TBTV isolates may be associated with lower p35 expression. Additionally, the migration of TB-JC p35 in gels remarkably differed from the wt (Fig. 2B), whereas the predicted molecular weight of p35 based on coding sequences is nearly identical in different TBTV isolates. It suggests that the post-translational protein modification of p35 in TB-JC may be different from that in other TBTV isolates. This differential modification of p35 in TB-JC might be from the phosphorylation process. Based on the prediction of GPS 3.0 (http://gps.biocuckoo.org/wsresult.php), four additional phosphorylation sites were identified at T46, T94, S97 and S133 in p35 of TB-JC compared with TBTV-Ch (Supplement Fig. 1)

**Figure 3:**
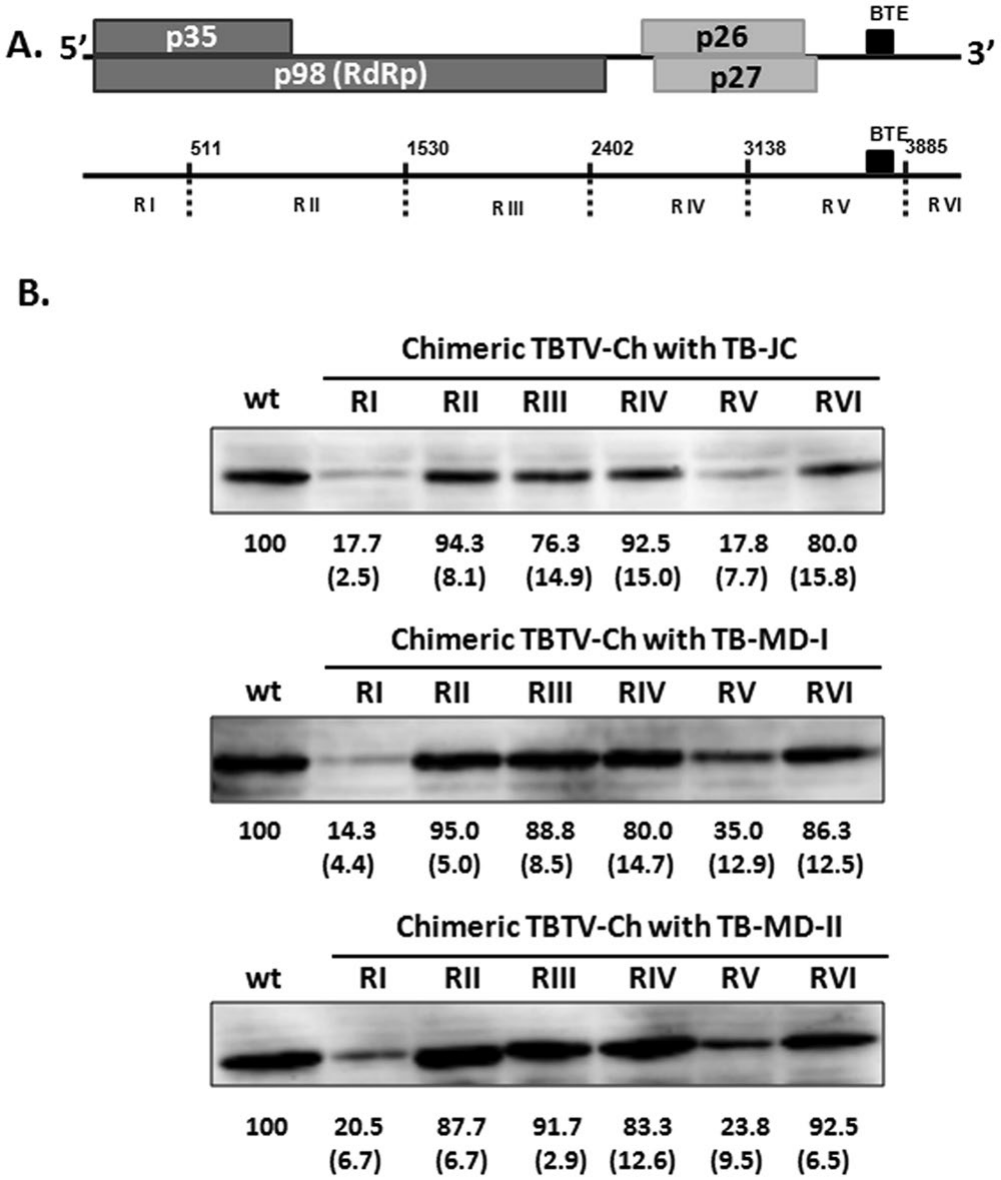
p35 expression in different types of chimeric TBTV. (**A**) Genome organization of TBTV and 6 chimeric regions. The BTE is located in the RV region. (**B**) p35 expression of wild type and chimeric TBTV-Ch. The basic vector is TBTV-Ch (wt), and RI to RVI was replaced with the corresponding region from TB-JC, TB-MD-I or TB-MD-II. The numbers under the western blots indicate the relative p35 level, and the numbers in brackets indicate the STEV.

For identifying the regions associated with the attenuated expression of p35 in mild TBTV isolates, TBTV genome was divided into six regions (RI to RVI) from 5′ to 3′ based on available single-cut enzyme sites in pTBTV-Ch. Local RNA domains such as BTE based on RNA structure prediction of TBTV were also taken into account. To map the core regions causing lower p35 expression, 18 chimeric TBTV clones were constructed through replacement of regions RI to RVI in TBTV-Ch with the corresponding regions from TB-JC, TB-MD-I and TB-MD-II (Fig. 3A). Chimeric TBTV-Ch containing RII, RIII, RIV, or RVI of the three new TBTV isolates exhibited only small decreases in the p35 level(76% to 95% of wt), whereas chimeric TBTV-Ch containing RI or RV from the new TBTV isolates was reduced to 14% to 35% of the wt level (Fig. 3B). This finding suggests that low level expression of p35 in these TBTV isolates is mainly caused by mutations within regions RI and RV.

### The RV region causes lower p35 expression in the mildly pathogenic TBTV isolates due to structure alterations of the BTE instead of specific sequences mutations within the BTE

**Figure 4:**
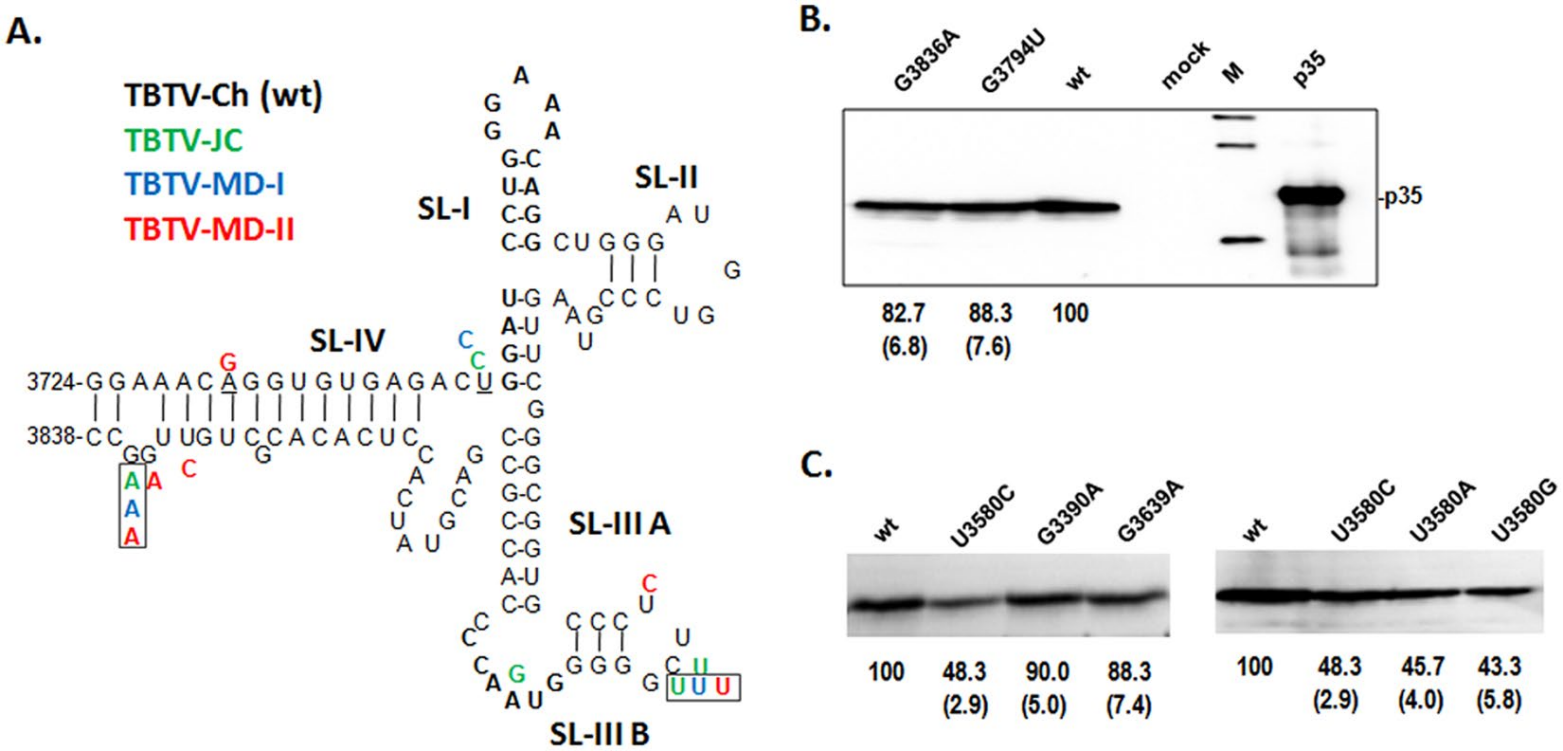
Effects of mutation sites within RV on p35 expression. (**A**) Mutation sites (colored) within the BTE region in other TBTV isolates compared with TBTV-Ch (wt). Framed nucleotides indicate the mutual mutation sites in TBTV-JC,TBTV-MD-I and TBTV-MD-II. (**B**) Effect of mutual mutation sites within BTE on p35 expression. (**C**) Effect of mutual mutation sites outside of BTE in RV on p35 expression. The numbers under the western blots indicate the relative p35 level, and the numbers in brackets indicate the STEV.

To determine why the RV region in TB-JC, TB-MD-I and TB-MD-II caused lower p35 expression, the RV region sequences were aligned to TBTV-Ch. Compared with TBTV-Ch, nine mutual variable positions (A3288G, A3339G, A3345G, G3390A, U3580C, G3639A, G3794U, G3836A, and G3852A) in RV regions of TB-JC, TB-MD-I and TB-MD-II were identified. The G3794U and G3836A mutations located within the BTE were separately introduced into TBTV-Ch (Fig. 4A). The G3794U and G3836A mutations caused slight decreases in the p35 levels (Fig. 4B), suggesting that low level of p35 expression in TB-JC, TB-MD-I and TB-MD-II was not primarily associated with mutual mutations in the BTE.

**Figure 5:**
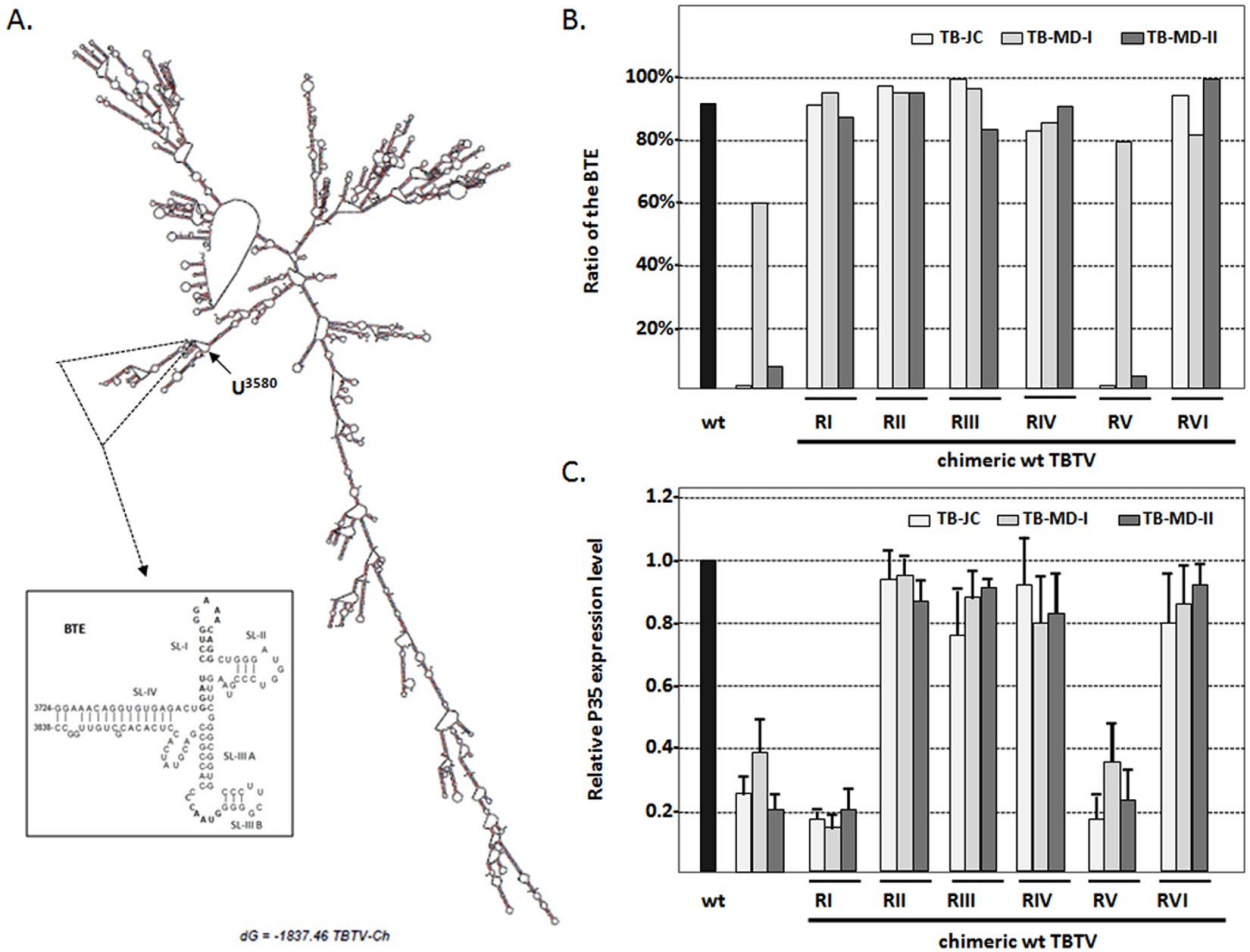
Relationship between the p35 expression level and ratio of the BTE in different TBTV constructs. (**A**) Secondary structure of full-length TBTV-Ch (wt) and the local secondary structure of the BTE. (**B**)Ratio of the BTE in the different TBTV constructs. (**C**) Relative p35 expression levels in the different TBTV constructs. Supporting data from Fig. 3B.

Next, U3580C, G3390A and G3639A locating upstream of the BTE within the RV region,, which have potential characteristic changing local structure, were individually introduced into TBTV-Ch. U3580C reduced p35 synthesis by ~50%, whereas G3390A and G3639A did not significantly decrease the p35 levels (Fig. 4C). Moreover, changing U3580 to either A or G (U3580A and U3580G) caused similar reductions in the p35 levels as U3580C (Fig. 4C). U3580 is located proximal to the base region of the BTE in the secondary structure of the TBTV genome (Fig. 5A); thus,U3580 mutations may decrease p35 translation by affecting the BTE structure.

To evaluate potential structural alterations of the BTE, secondary structure predictions of different TBTV constructs were conducted using Mfold^28^, and the percentages of full-length genome structures containing the BTE structure were determined (Supplement Table 2 and Fig. 5B).The ratios of TBTV-Ch, TB-JC, TB-MD-I and TB-MD-II containing BTE structure were 92%, 0%, 60% and 7%, respectively, suggesting a correlation with the relative p35 levels (100%, 25%, 38% and 20%, respectively)(Fig. 5B and C). Similarly, chimeric TBTV-Ch with RV from TB-JC, TB-MD-II and TB-MD-I had 0%, 4% and 80% structures containing BTE, which also suggested a correlation with the synthesized p35 level (18%, 24%, and 35%, respectively) (Fig. 5B and C). This finding suggests that mutations such as U3580C outside of the BTE may reduce the p35 level by affecting the BTE structure.

**Figure 6:**
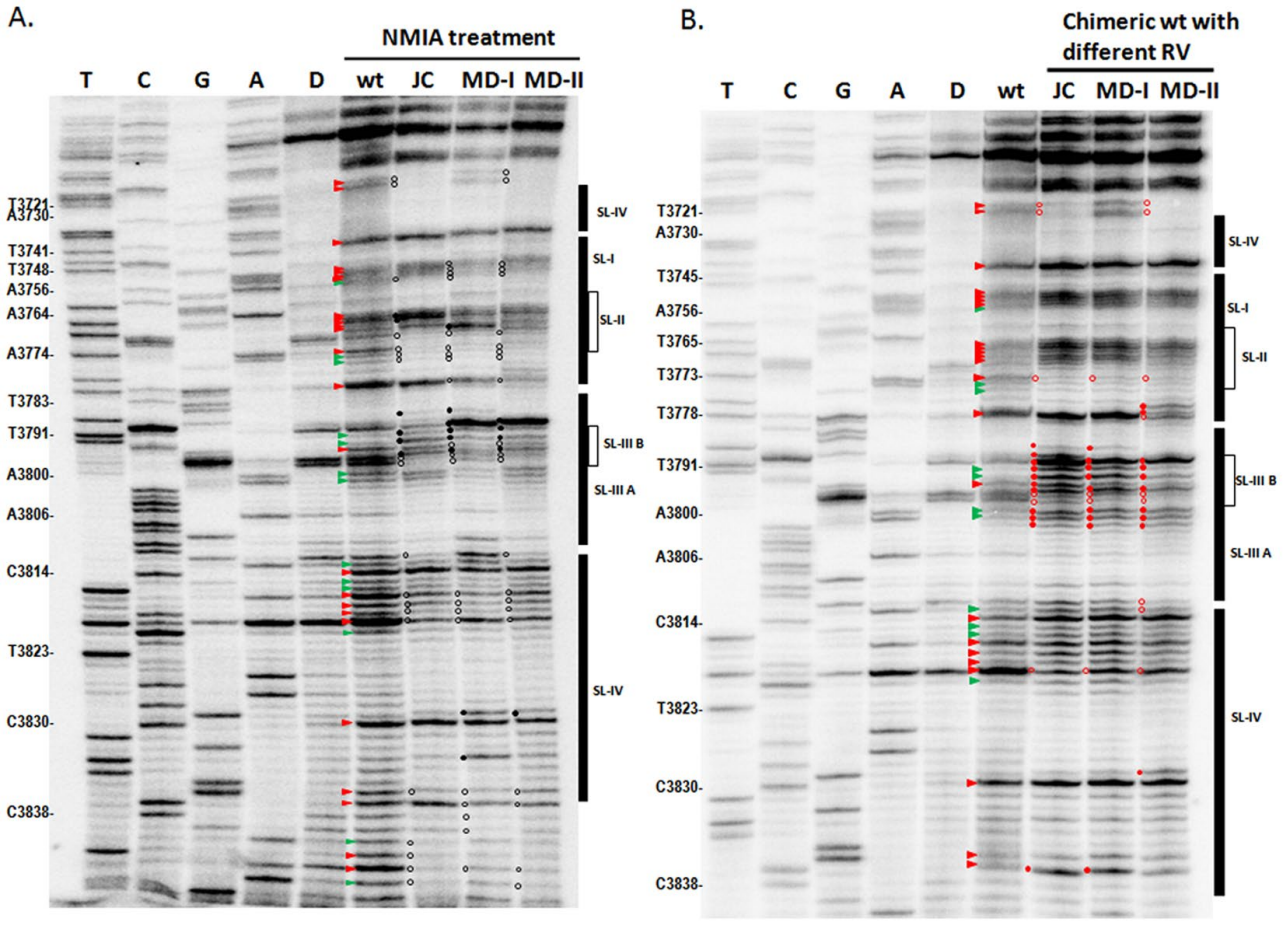
Structure probing of the BTE region in different TBTV isolates and chimeric TBTV using SHAPE. (**A**) SHAPE structure probing of the BTE region in TBTV-Ch and three new TBTV isolates. D:DMSO; wt:TBTV-Ch; JC:TBTV-JC; MD-I:TBTV-MD-I; MD-II:TBTV-MD-II. Red triangle: moderately high to high reactivity to NMIA compared with the DMSO control; green triangle: low to moderate reactivity to NMIA compared with the DMSO control. Black solid circle: higher NMIA activity in the new TBTV isolates compared with the wt; black empty circle: lower NMIA activity in the new TBTV isolates compared with the wt. (**B**) SHAPE structure probing of the BTE region in TBTV-Ch and chimeric TBTV with different RV regions. Red solid circle: higher NMIA activity in chimeric TBTV compared with the wt; red hollow circle: lower NMIA activity in chimeric TBTV compared with the wt.

**Figure 7:**
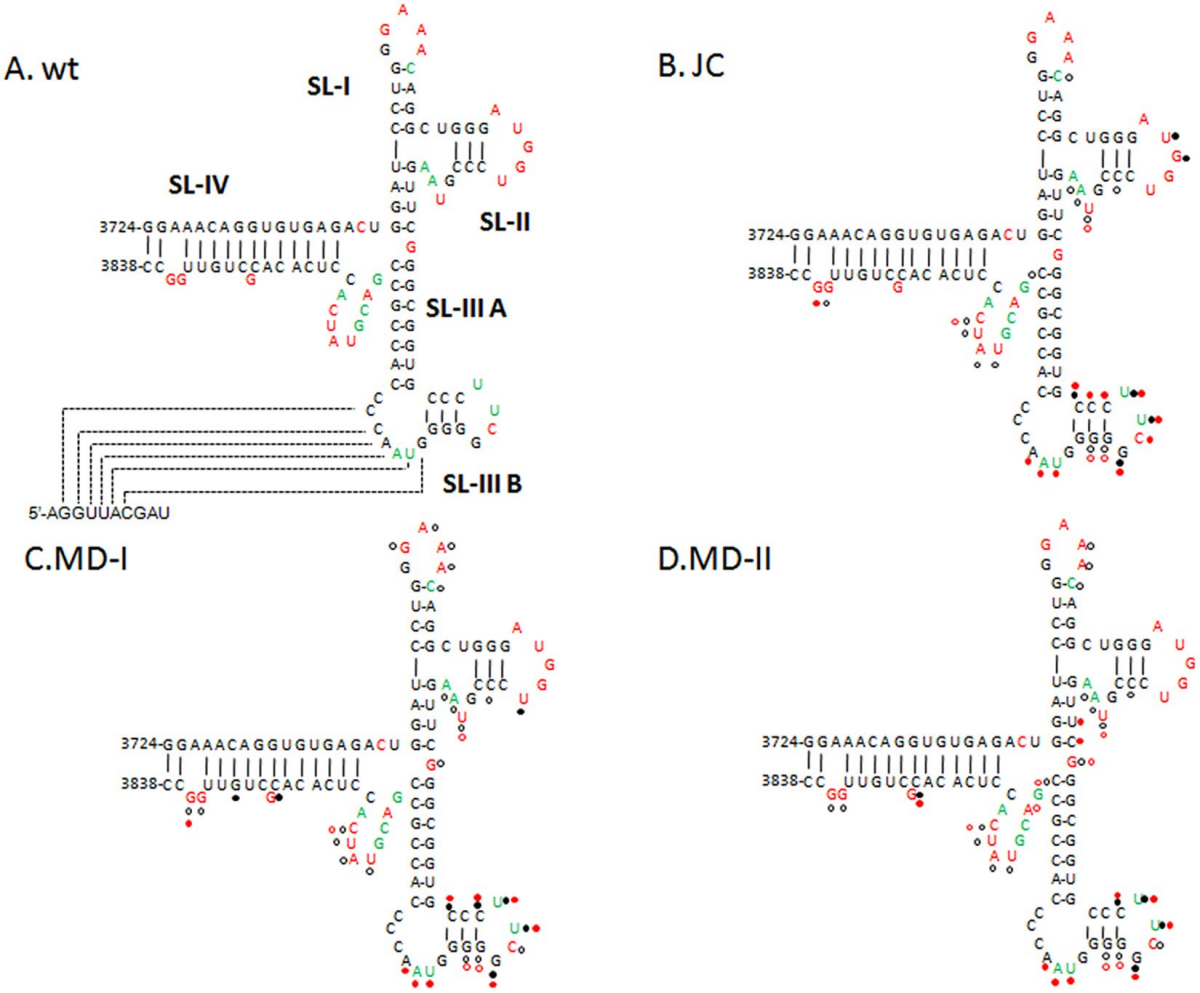
RNA structure of the BTE regions in different TBTV isolates and chimeric TBTV. (**A**) RNA structure of the BTE region in TBTV-Ch based on SHAPE data and Mfold. Red residues: moderately high to high reactivity to NMIA; green residues: low to moderate reactivity to NMIA; black residues: no reactivity to NMIA. (**B**), (**C**) and (**D**) RNA structure variations of the BTE regions in the three new TBTV isolates compared with the wt. Black solid circle: higher NMIA activity in the new TBTV isolates compared with the wt; black empty circle: lower NMIA activity in the new TBTV isolates compared with the wt. Red solid circle: higher NMIA activity in chimeric TBTV compared with the wt; red hollow circle: lower NMIA activity in chimeric TBTV compared with the wt.

To further evaluate the structure (including the long-distance RNA:RNA interaction) of the BTE region in different TBTV isolates, full-length TBTV gRNAs were subjected to RNA structure probing using selective 2-hydroxyl acylation and analyzed by primer extension (SHAPE). In TBTV-Ch (wt), the BTE region contained 5 stem-loops (labeled SL-I, SL-II, SL-III A, SL-III B and SL-IV; Figs 6A,B and 7A). The stem-loops, with the exception of SL-IIIA, contained flexible loop nucleotides that were reactive to NMIA (Figs 6AB and 7A). Nucleotides within the bulge loop of SL-IIIA lacked a SHAPE signal, possibly due to the long-distance RNA-RNA interaction between these residues and the 5′UTR (Figs 6A and 7A). In the new TBTV isolates, the BTE region had substantial differences in their SHAPE profiles, especially in the SL-IIIB region and SL-I loop (Figs 6A and 7B,C and D). Changes of SHAPE profiles in SL-I loop may affect the potential role of 17 nt conserved sequences, which is essential for BTE to regulate the translation. Changes of SHAPE profiles in SL-III B and SL-III A may affect the potential long-distance RNA-RNA interaction between BTE and 5′UTR, which is essential for the translation. Moreover, changes of SHAPE profile within the whole BTE may affect the structural stability of BTE, which is also important for the cap-independent translation. Based on the SHAPE profile of wt and mild TBTV isolates, it is suggested that the structure alteration of BTE region in mild TBTV isolates could affect three essential characteristic in BTE to attenuate the p35 expression. Similarly, chimeric TBTV-Ch with the RV region from the TB-JC, TB-MD-I and TB-MD-II isolates exhibited similar changes in the SHAPE profiles of the BTE regions (Figs 6B and 7B,C,D). These results suggest that decreased translation of p35 in the new TBTV isolates is associated with the structural but not sequence alterations in the BTE.

## Discussion

### Structural evolution of 3′ CITEs in different RNA viruses or different isolates of a given RNA virus

To date, several types of 3′ CITEs (TED, BTE, PTE, TSS,Y-Shape, I-Shape, and kl-TSS) have been reported in different RNA viruses possessing different structural characteristics^6, 8, 9, 17^. PEMV-2 even contains several types of 3′CITEs^17, 29, 30^. These structures regulate translation in a cap-independent manner by recruiting translation initiation factors or ribosome subunits^8, 9^ to their core elements. Additionally, similar acting model of 3′CITEs has been found in eukaryotic cells^10^. These findings indicated special evolution of the 3′ CITEs in different viral-derived and/or host-derived RNAs; however, the underlying structural evolutionary mechanism of the 3′CITEs in a given RNA virus remains unclear.

In this study, structural evolution of the BTE was identified in three mild TBTV isolates (Fig. 6), which were associated with lower p35 expression. This study is the first report of the structural evolution of 3′ CITEs (BTE in this study) in different isolates of a given RNA virus. Additionally, the structural alteration of the BTE in different TBTV isolates is not due to sequence mutations within the BTE region. BTE region sequences in TBTV-Ch and three new TBTV isolates were conserved with few mutations, which did not affect p35 expression(Fig. 4A and B). This result implies that a given RNA virus is able to monitor and maintain the sequence conservation of its 3′ CITE region during long term evolution since its 3′CITE is essential for the translation of viral proteins. Sequence conservation of essential elements may result from the viral quasispecies, which is mediated by the high mutation rate of the RNA-dependent RNA polymerase^19, 31^. Although sequences within the BTE region are conserved, TBTV may not monitor structural alterations of the BTE resulting from other nucleotide mutations outside the BTE region. This possibility also suggested the complexity of the local region surrounding the BTE. The complexity of the local region was also identified in the 3′ UTR region of *Turnip crinkle virus* (TCV), which contained an RNA-RNA interaction network mediated by Watson-Crick or non-Watson-Crick base pairing^32^.

In addition to the negative effects of structural alterations of the BTE on the p35 expression level, the RI region of the three mild TBTV isolates also contained mutations that attenuated p35 expression (Fig. 4). As shown in Fig. 1, the 5′ UTR plays essential roles in p35 expression through its long-distance interaction with the BTE (Fig. 1). The 5′ UTR sequences of the three mild TBTV isolates and TBTV-Ch were identical, whereas other RI region sequences in the three mild TBTV isolates contained many mutations compared with RI of TBTV-Ch(detailed data not shown). Special mutated sequences in the RI regions of the three mild isolates might form putative local interactions with the 5′ UTR and competitively inhibit the formation of long-distance interaction between the 5′ UTR and the BTE. This hypothesis also implied that the 5′ region of the TBTV genome might contain elements that regulate the formation or separation of the long distance between the 5′ UTR and the BTE, which regulates p35 translation. Modulation between the circular and linear forms of viral genomes during translation was identified in *Dengue virus* ^33^. For these mild TBTV isolates, the ratio of the linear form of the viral genome may be the majority, which is adverse to the cap-independent translation of p35.

### Mechanism of natural degeneration of tobacco bushy top disease

Over the past decade, tobacco bushy top disease has occurred through the sudden appearance of extreme virulence and degeneration in the Yunnan province of China^34^. In 2014, only 52 samples were detected to contain TBTV and TVDV from 269 diseased samples on Yunnan province^35^. Natural selection must play an essential role in pathogen evolution, although successful artificial management to prevent the transmission vector aphid in the field is important. TBTV and TVDV are the natural major pathogens of tobacco bushy top disease. In this study, three new mild TBTV isolates were isolated from Yunnan province. Their mild virulence should be partially associated with lower expression of both p35 (Fig. 2) and its -1 frameshift product (RdRp). Except the expression level of p35, phosphorylation modifications of p35 was different between TB-JC and TBTV-Ch (Fig. 2B; Supplement Fig. 1), which may affect the potential function of p35. Additionally, genome identity analysis of different TBTV isolates suggested that the RdRp coding region was most variable within the TBTV genome^34^. The RdRp encoded by different TBTVs may regulate the replication in different activities when the mutations are located in regions that determine essential amino acids. In addition to the changes in TBTV, we also found variable correlation between TBTV and TVDV that underwent natural selection^36^. CP encoded by TVDV mediated the natural transmission of TVDV and TBTV by aphids. However, at least two types of CP exist naturally. One type of CP is encoded by the TVDV which coexists with TBTV naturally, another type of CP encoded by the TVDV which does not coexist with TBTV^36^. Based on this study, it is suggested that low-level expression of p35, which is due to the structural alteration of BTE, was associated with the attenuated virulence of TBTV mild isolates. In addition, the attenuated virulence may be also associated with the translational-modification of p35 and the change of RdRp activity. Moreover, the change of aphid-transmission manner due to the mutation of TVDV CP could also affect the tobacco bushy top disease.

## Material and Methods

### DNA constructs

The pFluc-TB-5U plasmid was constructed by inserting the 5′ UTR (10 nt) of TBTV-Ch (NC004366) between the T7 promoter and firefly luciferase (Fluc)gene in the pT7-F-3′ UTRssp vector (a firefly luciferase reporter construct) using *Bam*H I and *Sma* I; the *in vitro* transcript from this plasmid was designated F-TB-5U. pFluc-TB-3U was constructed by inserting the 3′ UTR (645 nt) of TBTV-Ch (NC004366) downstream of the Fluc gene in the pT7-F-3′ UTRssp vector using *Pml* I and *Ssp* I; the *in vitro* transcript from this plasmid was designated F-TB-3U. pFluc-TB-5U3U contained the 5′ UTR and 3′ UTR of TBTV-Ch (NC004366) flanking the Fluc gene; the *in vitro* transcript of this plasmid was termed F-TB-5U3U. Mutant plasmids derived from pFluc-TB-5U3U were constructed by primer-mediated site-directed mutagenesis along with overlap extension PCR (detailed primer information is provided in Supplement Table 1), and the corresponding *in vitro* transcripts were designated F-TB-5Um3U, F-TB-5U3Um and F-TB-5Um3Um.

pTBTV-Ch was constructed by inserting the whole genome sequence of TBTV (NC004366) fused with the T7 promoter sequence into pMD18-T (Takara); the *in vitro* transcript from this plasmid was termed wt-TBTV in this study. Similar to pTBTV-Ch, pTBTV-JC, pTBTV-MD-I and pTBTV-MD-II contained the whole genome sequence of the JC (KM016224), MD-I (KM016225) and MD-II (KM067277) isolates of TBTV, respectively, and the corresponding *in vitro* transcripts were termed TB-JC, TB-MD-I and TB-MD-II.

All plasmids containing chimeric TBTV genomes were generated through digestion of PCR fragments amplified from TBTV-JC, TBTV-MD-I or TBTV-MD-II and replacement of the corresponding fragments in pTBTV-Ch using the corresponding enzyme sites. Mutant plasmids derived from pTBTV-Ch or other TBTV plasmids containing full-length genomes of TBTV isolates were constructed by primer-mediated site-directed mutagenesis along with overlap extension PCR. The primers used to generate the DNA constructs are listed in Supplement Table 1. All plasmids were verified by DNA sequencing.

### *In vitro* transcription

All plasmids containing full-length or mutant TBTV were linearized with *Eco*R V, and all luciferase reporter constructs containing the TBTV 5U and/or 3U fragments were linearized with *Ssp* I to make templates for RNA preparation. RNA was transcribed *in vitro* with the bacteriophage T7 RNA polymerase according to the manufacturer’s instructions (Promega, USA). The RNA integrity was verified by 1.0% agarose gel electrophoresis. The RNA concentration was determined by spectrophotometry.

### Protoplast inoculations and qRT-PCR

Protoplasts (5 × 10^6^ cells/ml) prepared by cellulose and pectinase digestion from BY-2^37^ were inoculated with 20 µg of different TBTV or genomic RNA transcripts as previously described^28^. Total RNA was extracted from protoplasts at 20 hpi and 40 hpi and subjected to qRT-PCR to evaluate the relative RNA accumulation of different TBTV constructs. The internal control for the qRT-PCR was elongation factor 1 alpha (EF1α); the fragments detected from TBTV were located at positions 700–970 and 1927–2170 using the primers shown in Supplement Table 1. At least three independent assays were performed for each constructs, and the relative expression levels and error bars was calculated by Microsoft Office Excel 2007 based on qRT-PCR raw data.

### *In vitro* translation

*In vitro* translation was assessed in wheat germ extract (WGE). RNA transcripts(1.6 pmol for TBTV and 0.4 pmol for the luciferase reporter vectors) were translated in a total reaction volume of 25 µl (12.5 µl of WGE, 2.0 µl of the amino acid mixture, 2.3 µl of 1 M potassium acetate, 0.53 µl of 0.1 M MgCl_2_, and 0.5 µl of RNase inhibitor) at 25 °C for 1.5 h as described by the manufacturer (Promega). To detect p35 from TBTV, the translated products were separated on an SDS-PAGE gel, probed with a p35 antibody^31^, detected with the ChampChemi^TM^ Top 420 Imager (Sage Creation Co. Ltd.) and quantified using the Quantity One one-dimensional analysis software (Bio-Rad). For the luciferase reporter RNA, the luciferase activity of the translated products was measured using the luciferase assay reporter system (Promega) in a GloMax^TM^ 20/20 luminometer (Promega). At least three independent assays were performed for each constructs, and the relative expression levels and error bars was calculated by Microsoft Office Excel 2007.

### RNA structural modeling-Mfold

The RNA secondary structures of TBTV or its derivatives were predicted with the RNA Folding Form (version 2.3 energies) from the Mfold web server (http://unafold.rna.albany.edu/?q=mfold/RNA-Folding-Form2.3)^38^. The folding temperature was 25 °C and other default settings were used.

### RNA structural probing-SHAPE

SHAPE structural probing of the TBTV BTE region was evaluated as previously described with some modifications^17^. Briefly, 25–50 pmol of TBTV gRNA was denatured at 95 °C for 3 min, snap-cooled on ice for 2 min and then incubated in SHAPE folding buffer (80 mM Tris-Cl, pH 8.0, 11 mM Mg(CH_3_COO)_2_, and 160 mM NH_4_Cl) at 37 °C for 20 min, followed by a subsequent 2 min on ice. Then, the folded RNA was treated with either 15 mM N-methylisatoic anhydride (NMIA) or the same volume of dimethyl sulfoxide (DMSO) as a negative control at 37 °C for 40 min. The RNA was recovered by ethanol precipitation and re-suspended in 0.5xTE buffer. Primer extension reactions were performed using [γ-^32^P] ATP-labeled oligonucleotides and the SuperScript III reverse transcriptase (Invitrogen) as previously described^39^. A primer(5-GAGAGGGAACCTCCAGAGT-3)complementary to positions 3866–3884 of TBTV was used for structural probing of the BTE region. The reaction products and ladders generated by Sanger sequencing were resolved on polyacrylamide gels containing 8 M urea. Then, the gels were dried and exposed to a phosphorimager screen, followed by detection with the Typhoon FLA-7000(GE Healthcare). The NMIA reactivity of each nucleotide was assigned as none, low to moderate and moderately high to high by visually inspecting the intensities of the individual bands compared with the DMSO reaction control. The RNA secondary structures were generated from the structure probing results combined with the Mfold predictions^38^. At least two independent SHAPE assays were performed for each constructs, and only reproducible effects are described.

## Electronic supplementary material

Supplement figure and tables
