# Supplementary material for: Structural alteration of a BYDV-like translation element (BTE) that attenuates p35 expression in three mild Tobacco bushy top virus isolates
Source: Sci Rep. 2017 Jun 23;7:4213. doi: 10.1038/s41598-017-04598-5 (PMC5482831; doi:10.1038/s41598-017-04598-5)
Supplement: Supplementary file 1 — Supplement figure and tables [file 41598_2017_4598_MOESM1_ESM.pdf]

Structural alteration of a BYDV-like translation element (BTE) that attenuates p35 expression in three mild *Tobacco bushy top virus* isolates

Deya Wang[1], Chengming Yu[1], Guolu Wang[1],Shanshan Liu[1], Kerong Shi[2], Xiangdong Li[1], Xuefeng Yuan[1,*]

([1]Department of Plant Pathology, College of Plant Protection, Shandong Agricultural University, Tai'an, 271018, P.R.China; [2]College of Animal Science and Veterinary Medicine, Shandong Agricultural University, Tai'an, 271018, P.R.China)

*Corresponding author, Tel:+86-538-8205608; E-mail address: snowpeak77@163.com (X.Yuan)

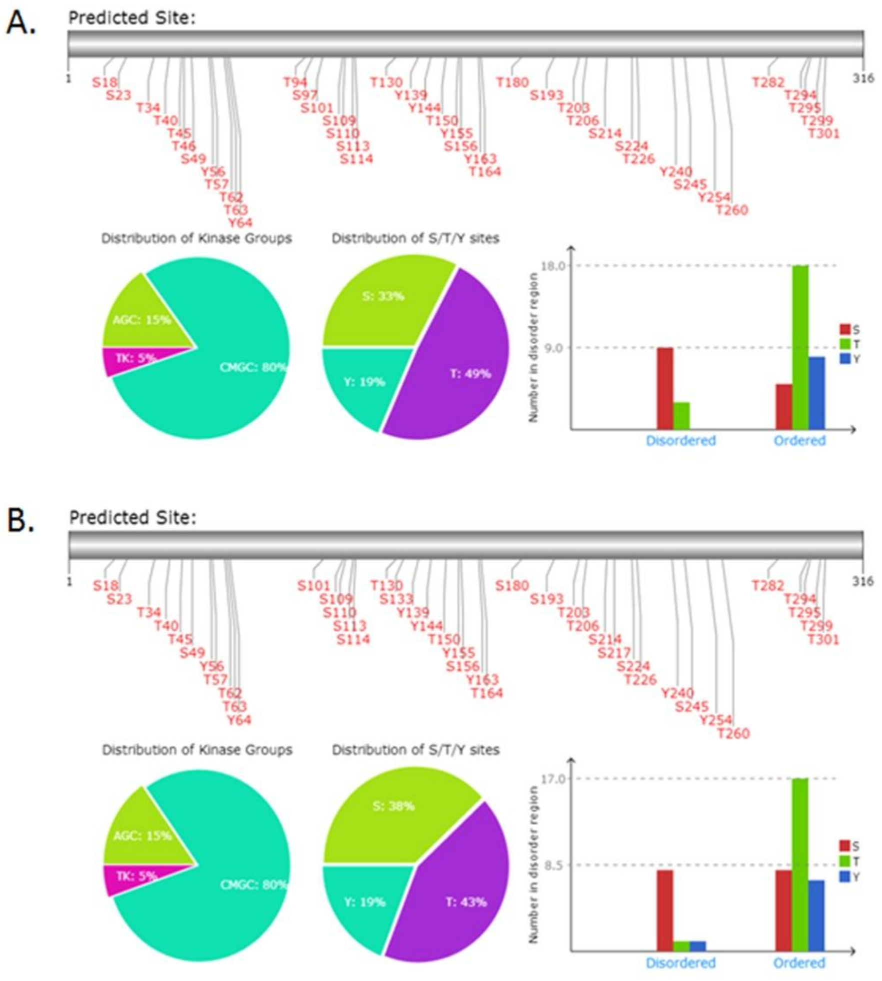

Supplement Figure 1. Prediction of the phosphorylation sites in p35 of TBTV using GPS 3.0.

(A) The potential phosphorylation sites in p35 of TB-JC.

(B) The potential phosphorylation sites in p35 of TBTV-Ch.

SUPPLEMENT TABLE 1 Primers used in this study

Primer name	Sequences (5'→3')	Purpose
TB-(1-10)-Fluc-F	GATCAGATCTAGGTTACGATCCCGGGGAGCTCATGGAAGACGCC	pF-5U
TB-(2-5)M- Fluc -F	GATCAGATCTA**ccaa**ACGATCCCGGGGAGCTCATGGAAGACGCC	pF-5Um
Fluc-(1154-1180)-R	GATCCTGGCATGCGAGAATCTGACGCAGGCA	pF-5U, pF-5Um
TB-(3508-3529)-F	GATCAATATTATTGGACACAAAACGGAGCTAG	pF-5Um
TB-(4136-4152)-R	GATCAATATTGGGCGCGAGAGAAAGTG	pF-3U, pF-5U3U
TB-(3800-3803)m-F	CTTCGGGGGT**ttgg**CCACC	pF-3Um
TB-(3800-3803)m-R	GGTGG**ccaa**ACCCCCGAAG	pF-3Um
TR-2402-F-BglII	ACAGATCTATGACTCATTGCCTGCTCCCA	mutants within BTE
TR-4152-R	ATGATATCGGGCGCGAGAGAAAGTGCTCATC	mutants within BTE
TB-(2-5)M-F	TACTGCAG*TAATACGACTCACTATA*GA**ccaa**ACGATATGGAGTTCATCAACA	pTB-5Um
TB-(2,5)M2-F	TACTGCAG*TAATACGACTCACTATA*GA**c**GU**a**ACGATATGGAGTTCATCAACA	pTB-5Um1
TB-721-R	GGGCTCACCAGCCACCTGC	pTB-5Um, pTB-5Um1
TB-BTE2m-F	GGGGGT**t**AC**g**CCACCGCCGACGTATCA	pTB-3Um1
TB-BTE2m-R	GGTGG**c**GT**a**ACCCCCGAAGGGCACC	pTB-3Um1
TB-BTE4m-F	GGGGGT**ttgg**CCACCGCCGACGTATCA	pTB-3Um
TB-BTE4m-R	GGTGG**ccaa**ACCCCCGAAGGGCACC	pTB-3Um
TB-BTE-M-1-F	TGGATCCTG**cc**t**tt**CAGGCTGGGATGGTCCC	pTB-BTm
TB-BTE-M-1-R	CTG**aaagg**CAGGATCCAGTCTCACACC	pTB-BTm
TB-BTE-Stem-1-F	TGTAAG**c**A**ttg**AGGTGTGAGACTGGATCCT	St-m1
TB-BTE-Stem-1-R	ACACCT**caa**T**g**CTTACACAGAGGTCTATCAACCA	St-m1
TBBTE-Stem1RM-F	GCT**caa**GG**g**CGATAAGCCGAGGTGTTGG	St-m1-com
TBBTE-Stem1 RM -R	ATCG**c**CC**ttg**AGCGTGTGAGGTGATACGT	St-m1-com
TB-BTE-Stem-2-F	GAAACAGG**acac**AGACTGGATCCTGGGAAAC	St-m2
TB-BTE-Stem-2-R	AGTCT**gtgt**CCTGTTTCCTTACACAGAGGT	St-m2
TBBTE-Stem2RM-F	ACCT**gtgt**CGCTGTTGGCCGATAAGC	St-m2-com
TBBTE-Stem2 RM -R	ACAGCG**acac**AGGTGATACGTCGGCGG	St-m2-com
TBTV-GDD-F	AACAAC**cgcgcagca**TGCATAGTTATATTTGACAGGCAGTACC	pTB-GDDm
TBTV-GDD-R	**tgctgcgcg**GTTGTTCATCAGTTCGTG	pTB-GDDm
EF1α-RT-F	TGGTGTCCTCAAGCCTGGTAT	qRT-PCR
EF1α-RT-R	ACGCTTGAGATCCTTAACCGC	qRT--CPR
TB-700-F	AGAGCAGGTGGCTGGTG	qRT-PCR
TB-970-R	CGACCACATCCCCTAGCAA	qRT-PCR
TB-1927-F	GACAGGCAGTACCTGTCCAC	qRT-PCR
TB-2170-R	CACATTCTCCAATGGCGTTCC	qRT-PCR
JC-T7-5'F-PstI	TACTGCAG*TAATACGACTCACTATA*GGGGTTACGATATG	JC,MDI,MDII-RI
TB-2561-R	GGGACAACGTAATCACT	JC,MDI,MDII-RII
TB-T7-221-F	*TAATACGACTCACTATA*GGTAGAAGAGGCTGTGCG	JC,MDI,MDII-RII
MDII-MluI-F	GCCACGCGTGCTGAGA	JC,MDI,MDII-RII, RIII
MDII-MluI-R	TCTCAGCACGCGTGGC	MDII-RI
TB-(1503-1522)-F	GCAGCTGGGTAAGCTTTACC	JC,MDI,MDII-RII, RIII
TB-(1503-1522)-R	GGTAAAGCTTACCCAGCTGC	JC,MDI,MDII-RII,RIII
TB-(3122-3138)-F	TTCCGATGAGCGGAGGG	JC,MDI,MDII-RIV, RV

TB-(3122-3138)-R	CCCTCCGCTCATCGGAA	JC,MDI,MDII-RIV, RV
TB-(3867-3885)-F	CTCTGGAGGTTCCCTCTCC	JC,MDI,MDII-RV, RVI
TB-(3867-3885)-R	GGAGAGGGAACCTCCAGAG	JC,MDI,MDII-RV, RVI
TB-G3390A-F	CCTAACCCT**a**ACTCAGGGG	G3390→A
TB-G3390A-R	CCCCTGAGT**t**AGGGTTAGG	G3390→A
TB-G3639A-F	CCCTTGGCA**a**GGGTGAT	G3639→A
TB-G3639A-R	ATCACCC**t**TGCCAAGGG	G3639→A
TB-T3580C-F	TGCGA**c**AACTGAGGTCGTC	U3580→C
TB-T3580C-R	GACGACCTCAGTT**g**TCGCA	U3580→C
TB-T3580A-F	AGTGCGA**a**AACTGAGGTCGT	U3580→A
TB-T3580A-R	ACGACCTCAGTT**t**TCGCACT	U3580→A
TB-T3580G-F	AGTGCGA**g**AACTGAGGTCGT	U3580→G
TB-T3580G-R	ACGACCTCAGTT**c**TCGCACT	U3580→G
TB-T7-3X(1-10)-F	TACTGCAG*TAATACGACTCACTATA*GGAGGTTACGATAGGTTACGATAGGTTA CGATATGGAGTTCATCAACAAGATAAAG	Triple-5U
TB-T7-2X(1-10)-F	TACTGCAG*TAATACGACTCACTATA*GAGGTTACGATAGGTTACGATATGGAGT TCATCAACAAGATAAAG	Double-5U
TB-T7-2X(1-10)m-F	TACTGCAG*TAATACGACTCACTATA*GAGGTTACGATA**c**GT**a**ACGATATG	5Um1+5U
TB-T7-3X(1-10)m-in-F	TACTGCAG*TAATACGACTCACTATA*GGAGGTTACGATAGGTTACGATA***c***GT***a***AC GATATGGAGTTCATCAACAAGATAAAG	Tri-5U-m1, Tri-5U-m1-com
TB-T7-3X(1-10)m-out-F	TACTGCAG*TAATACGACTCACTATA*GGA***c***GT***a***ACGATAGGTTACGATAGGTTAC GATATGGAGTTCATCAACAAGATAAAG	Tri-5U-m2, Tri-5U-m2-com

Note: T7 promoter: Italic; mutation sites: bold lowercase; enzyme sites: underlined

SUPPLEMENT TABLE 2 Summary of p35 expression level and ratio of BTE in different TBTV constructs

Constructs	Chimeric region	Donor of chimeric fragment	relative p35 level	Ratio of BTE
TBTV-Ch	N/A	N/A	100	46/50 (92.0%)
TB-JC	N/A	N/A	25.0 ± 5.0	0/49 (0%)
TB-MD-I	N/A	N/A	38.3 ± 10.4	18/30 (60.0%)
TB-MD-II	N/A	N/A	20.3 ± 5.0	3/43 (7.0%)
Chimeric TBTV-Ch	RI	TB-JC	17.7 ± 2.5	46/50 (92.0%)
	RII	TB-JC	94.3 ± 8.1	49/50 (98.0%)
	RIII	TB-JC	76.3 ± 14.9	50/50 (100%)
	RIV	TB-JC	92.5 ± 15.0	40/48 (83.3%)
	RV	TB-JC	17.8 ± 7.7	0/45 (0%)
	RVI	TB-JC	80.0 ± 15.8	46/48 (95.8%)
	RI	TB-MD-I	14.3 ± 4.4	48/50 (96.0%)
	RII	TB-MD-I	95.0 ± 5.0	48/50 (96.0%)
	RIII	TB-MD-I	88.8 ± 8.5	47/48 (97.9%)
	RIV	TB-MD-I	80.0 ± 14.7	43/50 (86.0%)
	RV	TB-MD-I	35.0 ± 12.9	38/47 (80.9%)
	RVI	TB-MD-I	86.3 ± 12.5	38/46 (82.6%)
	RI	TB-MD-II	20.5 ± 6.7	44/50 (88.0%)
	RII	TB-MD-II	87.7 ± 6.7	48/50 (96.0%)
	RIII	TB-MD-II	91.7 ± 2.9	33/39 (84.6%)
	RIV	TB-MD-II	83.3 ± 12.6	45/49 (91.8%)
	RV	TB-MD-II	23.8 ± 9.5	2/50 (4.0%)
	RVI	TB-MD-II	92.5 ± 6.5	33/33 (100%)

Note: RI: position 1-511; RII: Position 512-1530; RII:1531-2402; RIV:2402-3138; RV:3139-3885; RVI:3886-4152. N/A: not analyzed.
